# Aptamer-Functionalized Gold Nanoparticles for Drug Delivery to Gynecological Carcinoma Cells

**DOI:** 10.3390/cancers13164038

**Published:** 2021-08-11

**Authors:** Jessica Lopes-Nunes, Ana S. Agonia, Tiago Rosado, Eugénia Gallardo, Rita Palmeira-de-Oliveira, Ana Palmeira-de-Oliveira, José Martinez-de-Oliveira, José Fonseca-Moutinho, Maria Paula Cabral Campello, Artur Paiva, António Paulo, Alexa Vulgamott, Andrew D. Ellignton, Paula A. Oliveira, Carla Cruz

**Affiliations:** 1 CICS-UBI Centro de Investigação em Ciências da Saúde, Universidade da Beira Interior, Av. Infante D. Henrique, 6200-506 Covilhã, Portugal; jessicalonu@hotmail.com (J.L.-N.); tiagorosadofful@hotmail.com (T.R.); egallardo@fcsaude.ubi.pt (E.G.); rpo@fcsaude.ubi.pt (R.P.-d.-O.); apo@fcsaude.ubi.pt (A.P.-d.-O.); jmo@fcsaude.ubi.pt (J.M.-d.-O.); jafmoutinho@fcsaude.ubi.pt (J.F.-M.); 2 Labfit-HPRD Health Products Research and Development, Lda, Edifício UBIMEDICAL Estrada Municipal 506, 6200-284 Covilhã, Portugal; sofiagonia@gmail.com; 3 C4-Cloud Computing Competence Centre, UBIMedical, Universidade da Beira Interior, EM506, 6200-284 Covilhã, Portugal; 4 Center for Neuroscience and Cell Biology, University of Coimbra, Universidade de Coimbra, Rua Larga, 3004-504 Coimbra, Portugal; 5 Quinta do Alvito, Centro Hospitalar Universitário Cova da Beira, 6200-251 Covilhã, Portugal; 6 Centro de Ciências e Tecnologias Nucleares, Instituto Superior Técnico, Universidade de Lisboa, Estrada Nacional 10, 2695-066 Bobadela LRS, Portugal; pcampelo@ctn.tecnico.ulisboa.pt (M.P.C.C.); apaulo@ctn.tecnico.ulisboa.pt (A.P.); 7 DECN-Departamento de Engenharia e Ciências Nucleares, Instituto Superior Técnico, Universidade de Lisboa, Estrada Nacional 10, 2695-066 Bobadela LRS, Portugal; 8 Unidade de Gestão Operacional em Citometria, Centro Hospitalar e Universitário de Coimbra (CHUC), 3000-075 Coimbra, Portugal; artur.paiva@chuc.min-saude.pt; 9 CIMAGO/iCBR/CIBB, Faculdade de Medicina da Universidade de Coimbra, 3000-370 Coimbra, Portugal; 10 Ciências Biomédicas Laboratoriais, Instituto Politécnico de Coimbra, ESTESC-Coimbra Health School, 3046-854 Coimbra, Portugal; 11 Department of Molecular Biosciences, The University of Texas at Austin, Austin, TX 78712, USA; avulgamott@utexas.edu (A.V.); ellingtonlab@gmail.com (A.D.E.); 12 Centre for Research and Technology of Agro Environmental and Biological Sciences (CITAB), Inov4Agro, University of Trás os Montes and Alto Douro (UTAD), Quinta de Prados, 5000-801 Vila Real, Portugal; pamo@utad.pt

**Keywords:** cervical cancer, endometrial carcinoma, acridine orange derivative, Imiquimod, aptamer, gold nanoparticle

## Abstract

**Simple Summary:**

Gynecologic cancers are a major concern since they can significantly affect women’s lives. Moreover, the available therapeutic options increase the risk of infertility. Thus, better treatment options for women with these pathologies represent an urgent need. The DNA G-quadruplex aptamer AS1411 covalently conjugated to gold nanoparticles presents high selectivity to cancer cells and can be used as a drug delivery system for anticancer drugs. By using these AS1411-functionalized nanoparticles we aimed to improve the anticancer effect of two molecules with promising effects that can be improved. After the supramolecular conjugation of AS1411-gold nanoparticles with the molecules, we were able to overcome the lack of selectivity in the case of a potential anticancer ligand (C_8_) and improve the anticancer effect of a commercially available drug (Imiquimod). Overall, with the resulting nanoparticles the benefits of both drugs were significantly improved in gynecologic cancer cells.

**Abstract:**

Cervical cancer is one of the most common cancers and is one of the major cause of deaths in women, especially in underdeveloped countries. The patients are usually treated with surgery, radiotherapy, and chemotherapy. However, these treatments can cause several side effects and may lead to infertility. Another concerning gynecologic cancer is endometrial cancer, in which a high number of patients present a poor prognosis with low survival rates. AS1411, a DNA aptamer, increases anticancer therapeutic selectivity, and through its conjugation with gold nanoparticles (AS1411-AuNPs) it is possible to improve the anticancer effects. Therefore, AS1411-AuNPs are potential drug carriers for selectively delivering therapeutic drugs to cervical cancer. In this work, we used AS1411-AuNPs as a carrier for an acridine orange derivative (C_8_) or Imiquimod (IQ). The AS1411 aptamer was covalently bound to AuNPs, and each drug was associated via supramolecular assembly. The final nanoparticles presented suitable properties for pharmaceutical applications, such as small size, negative charge, and favorable drug release properties. Cellular uptake was characterized by confocal microscopy and flow cytometry, and effects on cellular viability were determined by MTT assay. The nanoparticles were then incorporated into a gel formulation of polyethylene glycol, suitable for topical application in the female genital tract. This gel showed promising tissue retention properties in Franz cells studies in the porcine vaginal epithelia. These findings suggest that the tested nanoparticles are promising drug carriers for cervical cancer therapy.

## 1. Introduction

Cervical cancer is the most common human papillomavirus (HPV)-associated cancer among women [1,2,3], and is mainly caused by high-risk HPVs 16 and 18 [4]. In recent years, the combination of screening programs and vaccination against HPV has greatly reduced cervical cancer incidence and mortality [4]. However, the populational coverage remains low, and for women already infected, the vaccination will not cure them or treat precancer lesions. For cervical dysplasia, the therapeutic options available include surgery, radio and chemotherapy, increasing the risk of infertility [5].

Moreover, endometrial cancer is the most common gynecologic cancer, with an increasing incidence worldwide. Patients that are diagnosed at an early stage present good prognosis and high survival rates, although when diagnosed at a late stage they have poor prognosis, mostly caused by tumor metastasis, which has low survival rates and limited treatment options [6,7,8,9]. Thus, improved treatment options for women that already present cervical and endometrial cancer are still necessary.

Aptamers are structured nucleic acids that have several advantages relative to antibodies, including chemical synthesis and stability, conformational flexibility, and an overall smaller size [10]. Some aptamers have guanine (G)-rich tracts that can form G-quadruplex (G4) structures [11]. AS1411 is a G4 DNA aptamer that exhibits high affinity for nucleolin, has antiproliferative properties, and induces apoptosis in cancer cells with reduced toxicity in normal ones [12,13,14,15]. Its biological effects are mainly caused by cell cycle arrest, inhibition of NF-κB signaling, induction of tumor suppressor gene expression, and reduction of bcl-2 expression [12]. Indeed, AS1411 reached Phases 1 and 2 clinical trials [16,17] but demonstrated low potency (µM) and suboptimal pharmacology (rapid clearance from the body) [16,18]. To improve its malignant cell selectivity/accumulation and to increase its toxicity, AS1411 has been associated with nanoparticles, such as liposomes and gold nanoparticles (AuNPs) [18,19,20,21,22]. Previously, Malik et al. reported that AS1411 bound to AuNPs (AS1411-AuNPs) had higher cellular uptake and improved antiproliferative/cytotoxic effects relative to unconjugated AS1411 or control sequences [21]. These aptamer-conjugated nanoparticles could deliver ligands efficiently and selectively to cancer cells [21,23]. 

**Figure 1:**
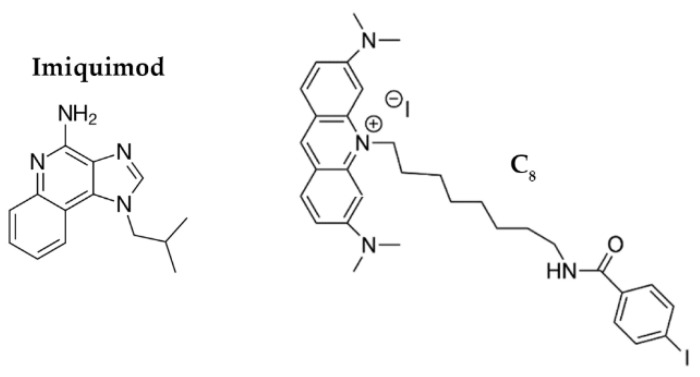
Chemical structures of Imiquimod and 10-(8-(4-iodobenzamide) octyl)-3,6-bis (dimethylamine) acridinium iodide (C_8_).

Since conjugation of the aptamer to the AuNPs improves cellular uptake, we hypothesized that AS1411-AuNPs may also be a promising drug carrier for cancer cells, taking advantage intrinsic anticancer potential/selectivity of the AS1411-AuNPs to improve the effects of some drugs. To study this possibility, we focused on Imiquimod (IQ; Figure 1) and acridine orange derivative 10-(8-(4-iodobenzamide) octyl)-3,6-bis (dimethylamine) acridinium iodide (C_8_; Figure 1). 

IQ is an imidazoquinoline that can modulate the immune response through TLR7/8 activation, increase the activation of the NF-κB signaling pathway, and cause proapoptotic activity by triggering the caspase pathway [24]. It has proven anticancer and antiviral effects [25], but its biological properties are sub-optimal. It is currently used in premalignant lesions caused by low-risk HPV infections [26]. It has also been tested in patients with recurrent high-grade vulvar, vaginal or cervical intraepithelial neoplasia and demonstrated to be a potential anticancer drug for these situations [27]. 

Similarly, C_8_ is an acridine orange derivative that exerts anticancer effects. However, this ligand has high toxicity in both malignant and normal cell lines [28]. C_8_ can bind to nucleolin-targeted aptamers, and it can potentially stabilize G4 structures. Thus, it may be possible to add C_8_ to AS1411 to improve its therapeutic index while also enhancing an overall supramolecular assembly with nanoparticles [23,29,30].

Herein we describe the synthesis and characterization of AS1411-AuNPs and their potential as drug carriers to selectively deliver IQ and C_8_ to nucleolin-positive cancer cells. The nanoparticles were characterized by different physico-chemical methods and the drug release kinetics assessed. Furthermore, their anticancer activity, cellular uptake and lysosomal trapping were evaluated. Finally, their potential to be incorporated in a gel formulation and their vaginal penetration profile was studied.

## 2. Materials and Methods

### 2.1. Oligonucleotides, Ligands and AuNPs

AS1411 was purchased from Eurogentec (Liège, Belgium) with HPLC-grade purification and a purity of 98%. The oligonucleotide had a regular DNA backbone (i.e., phosphodiester) and a 5′-Thiol C6 S-S modification (Thio-MC6-D). Stock solutions of 500 µM were prepared using Milli-Q water. Light-on LysoView™ 540 was purchased from Biotium (San Francisco, CA, USA), Hoechst 33342 from Thermo Scientific (Waltham, MA, USA) and IQ from Tokyo Chemical Industry (Zwijndrecht, Belgium). The ligands, C_8_ [31] and IQ were dissolved in DMSO to obtain a 10 mM and 5 mM stock solution, respectively, and further diluted in milli-Q water. Stabilized AuNPs (5 nm diameter) suspension in citrate buffer was purchased from Sigma-Aldrich (St. Louis, MO, USA) at concentration of 5.5 × 10^13^ particles/mL. 

### 2.2. Nanoparticles Synthesis

Functionalization of gold nanospheres with thiolated AS1411 was achieved using a method adapted from the one previously described in Malik et al. [21] based on the original protocol of Mirkin et al. [32]. This methodology was used since, the resulting nanoparticles were efficiently internalized and showed selectivity for cancer cells compared to non-malignant cells. Moreover, by using an in vivo model, the authors demonstrated that systemic administration of AS1411-AuNPs could completely inhibit tumor growth with no signs of toxicity [32]. Since Malik et al. reached such interesting results, we decided to use a similar approach to improve the potential of IQ and C_8_ and to increase the selectivity of this drugs towards malignant cells.

Thiolated oligonucleotides were first reduced by incubating with a filtrated solution of 10 mM tris-(2-carboxyethyl)-phosphine hydrochloride (TCEP, Pierce, Waltham, MA, USA; in a 100× molar excess) for 6 h at room temperature. Then, the oligonucleotides were added to 5 nm colloidal gold nanospheres to give a final oligonucleotide:gold nanoparticle ratio of 200:1. After overnight incubation on a rotator, filtrated solution of 20 mM KPi was added until reaching a final concentration of 10 mM. Four hours later, filtrated 3 M NaCl was added to the solution until reaching a final concentration of 100 mM and the solution was left in rotation for more 6 h. To finish, more filtrated NaCl solution was added (until reaching a final concentration of 300 mM) and it was incubated in rotation, at room temperature for an additional overnight.

The sample was inserted in a D-tube Dialyzer Maxi (Milipore, Burlington, MA, USA; MWCO 3.5 KDa). To remove the salts from the solution of aptamer functionalized AuNPs, dialysis was performed for 48 h and the H_2_O Milli Q was replaced two times a day. 

Supramolecular assembly of C_8_ or IQ with AS1411-AuNPs was obtained after mixing both components. In case of AS1411-AuNPs with C_8_ the mixture was performed with a molar ratio of 15:1 and for AS1411-AuNPs with IQ with a molar ratio of 15:5. 

### 2.3. Nanoparticles Characterization

Hydrodynamic size analysis and zeta potential of the aptamer-modified AuNPs were determined using Zetasizer Nano ZS equipment (Malvern Instruments, Worcestershire, UK). Particles were diluted in purified water for these measurements.

Additionally, the morphology was assessed through transmission electron microscopy (TEM) using a Hitachi-HT7700 (Krefeld, Germany). Samples were prepared by placing a drop of particles dispersed in purified water on nickel grid coated with carbon and allowing the samples to dry for 15 min. Images were captured at an accelerating voltage of 80 kV and with magnification of 100 k.

### 2.4. Drug Release Kinetics Assay

Initially, a standard fluorescence curve was established with different concentrations of C_8_ solution in order to enable the determination of the exact amount of drug released by the AS1411-AuNPs, using the equipment Spectra MAX GEMINI EM (Molecular Devices, San Jose, CA, USA).

For drug release kinetics assay, 100 µL of AS1411-AuNPs with C_8_ at a final concentration of 5 µM were inserted in Slide-A-Lyzer (Thermo Fisher Scientific, Waltham, MA, USA; MWCO of 3.5 KDa). The dialysis device was inserted in an Eppendorf with 1 mL of buffer (20 mM KPi and 100 mM KCl) and, at different time points (0 min, 5 min, 15 min, 30 min, 1 h, 2 h, 3 h, 4 h, 5 h, 6 h, 12 h, 24 h and 48 h)., 100 µL of buffer solution was collected and, subsequently, replaced. The samples were in agitation under room temperature.

The fluorescence of the collected buffer (λ_ex_ = 498 nm and λ_em_ = 530 nm) was measured, and the obtained value in each range was used to estimate the percentage of drug released.

### 2.5. Confocal Fluorescence Microscopy

For fluorescence confocal microscopy assays, human cervical cancer cells (HeLa; ref. CCL-2™, ATCC), human endometrial carcinoma cells (HEC-1-A; ref. HTB-112™, ATCC), and normal human dermal fibroblasts (NHDF; ref. PCS-201-012™, ATCC) were seeded at 5 × 10^4^ cells/mL in a treated µ-slide 8 well (IBIDI, Gräfelfing, Germany) in 200 µL of medium and grown at 37 °C under a 95% air and 5% CO_2_ humidified atmosphere. 

For the medium replacement assay, performed to determine if the nanoparticles are retained in the cell lines, HeLa, HEC-1-A and NHDF cells were treated with AS1411-AuNPs with IQ or C_8_. The day after, medium was replaced by new one without any treatment. Nuclei were stained with the 1 µM nuclear probe Hoechst 33342 for 15 min. The probe was washed off by rinsing with PBS three times and cells were imaged using a Zeiss AxioObserver LSM 710 microscope (Oberkochen, Germany).

For the LysoViewTM experiments, cells were treated with the nanoparticles assembled with IQ or C_8_ for 2 h. The cells were then washed three times with PBS and nuclei were stained with 1 µM of nuclear probe Hoechst 33,342 for 15 min. After that, the probe was washed off by rinsing with PBS three times. Then, LysoView was added to the wells and it was left for 30 min. The cells were washed three times with PBS and imaged using a Zeiss AxioObserver LSM 710 microscope with 405, 488 and 555 nm laser excitation for Hoechst 33342, drug (IQ or C_8_) and LysoViewTM 540, respectively.

For nucleolin immunocytochemistry, HeLa and HEC-1-A cells were firstly incubated with the nucleolin antibody (1:100) during 2 h and a secondary antibody (Alexa Fluor 647^®^, 1:1000) for 1 h. Nuclei were stained with the 1 µM nuclear probe Hoechst 33,342 for 15 min. The probe was washed off by rinsing with PBS three times. Then, cells were incubated with AS1411-AuNPs with IQ or C_8_ for 15 min, washed three times with PBS and then imaged using a Zeiss AxioObserver LSM 710 microscope. 

### 2.6. Flow Cytometry

HeLa, HEC-1-A and NHDF cells were seeded in 12-well plates at a density of 50 × 10^4^ cells/well and incubated overnight for cell adhesion. Then, cells were treated for 24 h with the AS1411-AuNPs with C_8_ and free C_8_ as control. After the incubation period, the wells were washed by rinsing with PBS three times and cells were then trypsinized, resuspended in PBS and analyzed in a BD FACSCanto™ II flow cytometry system (BD Life Sciences, Franklin Lakes, NJ, USA) to evaluate the uptake of the aptamer-ligand complexes. C_8_ fluorescence was used to monitor the cellular uptake of AS1411-AuNPs. Non-specific staining, doublets and debris were excluded. 

### 2.7. Cytotoxicity Assay

HeLa cancer cells were grown in DMEM medium supplemented with 10% fetal bovine serum and a 1% streptomycin–penicillin antibiotic. NHDF and HEC-1-A cells were grown in RPMI medium, supplemented with 10% fetal bovine serum (FBS), 1% streptomycin–penicillin antibiotic, 0.01 M HEPES, 0.02 M L-glutamine and 0.001 M sodium pyruvate. Upon confluence, cells were seeded into 48-well culture plates (2 × 10^4^ cells/mL) in 250 µL of medium. Cells were treated with 200 µL of medium with different stimuli. Firstly, IC_50_ of IQ or C_8_ in the cell lines was determined. Then, cells were treated with different conditions: control, AS1411-AuNPs (15 µM), AS1411-AuNPs with IQ (15 µM:5 µM) or AS1411-AuNPs with C_8_ (15 µM:1 µM).

After 3 days of incubation under a controlled humidified atmosphere at 37 °C and 5% CO_2_, 50 μL of 5 mg/mL thiazolyl blue tetrazolium bromide (MTT) solution was added into each well and were further incubated for 1 h. The resulting formazan crystals were dissolved in 250 μL of DMSO and the optical density (OD) was recorded at 570 nm. Cell viability was determined using wells without compounds as the control.

### 2.8. Franz Cell Experiments

For the Franz cell experiments, the AS1411-AuNPs with C_8_ were incorporated in two different topical formulations. The difference between them was the propylene glycol percentage (8% or 10%). Gels were prepared by dissolving potassium sorbate (0.14% *w*/*w*, used as preservative) in sterile water (90.36% or 88.36%) and further dispersing 1.5% (*w*/*w*) of hydroxypropylmethyl cellulose (Methocel K100, DOW Chemical Company, Midland, MI, USA) using a helical stirrer (Heidolph RZR 2041, Heidolph Instruments GmbH & Co., Schwabach, Germany). Propylene glycol (8% or 10% *w*/*w*) was added under agitation. After the gel preparation, the nanoparticles (7.5 µM AS1411-AuNPs with 0.5 µM C_8_) were added.

Tissue permeation studies were performed in Static Franz Diffusion Vertical Cells (Model V6A-02, SES GMBH, Waldbrunn, Germany). The receptor chamber was filled with PBS (pH 7.4) up to 5 mL and magnetic stirring was maintained at 500 rpm at 37 °C. The diffusion area was 1.77 cm2 and on the donor chamber about 300 mg of each formulation was applied (weighted on an analytical balance on pre-filled syringes, before and after application on the chamber to determine the exact amount dispensed and available for diffusion). The interface between the two chambers was a vaginal porcine tissue and the total experiment time was 24 h, with samples (200 µL) being collected at different time points (0, 6, 12 and 24 h). The volume collected was replaced by pre-heated fresh PBS. Samples were quantified by high-performance liquid chromatography coupled to fluorescence detector (HPLC-FLD), described in the “Study of the Release Kinetics of C_8_” section.

The vaginal tissue was processed in order to quantify the C_8_ present in the sample. For that, the porcine vaginal tissue was sonicated in PBS (2 µL of PBS per mg of tissue) and the supernatant was collected and analyzed. Each formulation was tested in three different tissues (from different animals) in experiments performed in three independents days (*n* = 3).

Vaginal tissues used throughout the experiments were obtained from a local slaughterhouse (pigs up to 6 months old). The organs were transported to the lab and processed within 3 h from time of collection. The vagina was isolated with surgical scissors, opened longitudinally, washed with normal saline and wrapped in aluminum foil before being stored in zipped plastic bags, at −20 °C, for no more than 7 months. Immediately before the experiments, tissues were thawed in normal saline. Portions of epithelial vaginal tissue with controlled thickness were obtained by horizontally slicing the vagina with a manual dermatome up to a maximum of 300 µm (measured with an electronic digital micrometer (Vogel, model 231061).

### 2.9. Study of the Release Kinetics of C_8_

A simple HPLC-FLD method was developed and validated according to the guiding principles of the Food and Drug Administration (https://www.fda.gov/regulatory-information/search-fda-guidance-documents/analytical-procedures-and-methods-validation-drugs-and-biologics (accessed on 15 September 2020)). The assays were performed using a 1290 HPLC with a binary pump coupled to a 1260 FLD detector from Agilent Technologies (Soquimica, Lisbon, Portugal). For compound separation, a YMC-Triart PFP (5 μm, 4.6 i.d. × 150 mm) analytical column coupled to a Guard-c holder (4 × 10 mm) and a Triart PFP (5 μm, 3 × 10 mm) pre-column were used, all from YMC Europe GMBH (Solítica, Lisbon, Portugal). The mobile phase consisted of methanol/0.1% trifluoroacetic acid (9:1, *v*/*v*), operated in the isocratic mode with a flow of 0.5 mL/min, and the injection volume was 50 μL. The column and autosampler temperatures were set at 25 and 4 °C, respectively, and the analyte was detected at 498 and 530 nm as excitation and emission wavelengths, respectively. A calibration curve between 0.98 and 500 ng/mL was prepared with several successive dilutions of the analyte, using the mobile phase as solvent. Figure S1 presents a chromatogram (the retention time of the analyte was 9.57 min).

### 2.10. Statistical Analysis

All data are expressed as mean ± SEM of at least three independent experiments. Statistical analysis was performed using unpaired two tailed Student’s *t* test. Values of *p* < 0.05 were considered significant. All statistical analysis was made using the GraphPad Prism 6.0 Software (GraphPad Sotware Inc, San Diego, CA, USA).

## 3. Results and Discussion

Citrate AuNPs were functionalized with thiolated AS1411 through a methodology based on increasing concentrations of salts. Then, AS1411-AuNPs were mixed with C_8_ (molar ratio of 15:1) and IQ (molar ratio of 15:5). These ratios were defined in accordance with a previous report [23], in which it was demonstrated that due to the binding strength of C_8_ to AS1411, with this molar ratio there is no free C_8_ in the solution, and with the IC_50_ of each drug determined after 3 days of incubation (Figure S2). Additionally, the molar ratios were chosen taking into account the in vitro experiments, in order to have the highest concentration to elicit significant cell toxicity and still suitable to observe any changes induced by the ligand complexation to the aptamer, as previously reported [23].

**Figure 2:**
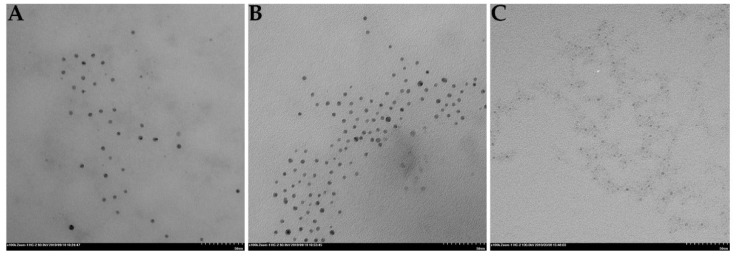
TEM images of (**A**) AS1411-gold nanoparticles, (**B**) AS1411-gold nanoparticles with IQ, and (**C**) AS1411-gold nanoparticles with C_8_.

The morphology of these aptamer-functionalized nanoparticles was analyzed by transmission electron microscopy (TEM) (Figures S3 and S4). As observed in Figure S3 and Figure 2, the nanoparticles present a uniform size (Figure S4) and are not aggregated. When IQ and C_8_ were added, the nanoparticles with IQ (Figure 2B) and with C_8_ (Figure 2C) also presented a uniform size (Figure S4) and distribution, without any aggregation, as previously observed. In these images, the metallic core of the resulting nanoparticles can be observed (Figure 2).

Dynamic light scattering (DLS) was performed to assess the size variation in each step of assembly, as presented in Table 1. As expected, the starting AuNPs (citrate AuNPs) showed the smallest size. When AS1411 was attached to the surface of AuNPs an increment in size was obtained, from a hydrodynamic diameter of 12.00 ± 1.20 nm to 15.76 ± 1.64 nm. Finally, the hydrodynamic diameter increased to values ranging between 18.30 and 21.16 nm, when C_8_ and IQ were supramolecularly associated with aptamer-functionalized AuNPs. Due to their small size, these nanoparticles, aside from the active targeting resulting from the coating with the AS1411 aptamer, also benefit from the enhanced permeability and retention (EPR) effect [33].

Additionally, the Zeta potential of the particles was measured, and we verified that all of the nanoparticles presented a negative charge in the range of −24.00 to −41.30, and we observed more negative values for the AuNPs carrying the anti-cancer compounds. Since the surfaces of blood vessels and cells are negatively charged components, these nanoparticles can avoid nonspecific interactions [34]. However, the G4 aptamer, which is more densely negatively charged than duplex DNA, can favor the interaction with positively charged pockets of proteins [15], namely nucleolin.

**Figure 3:**
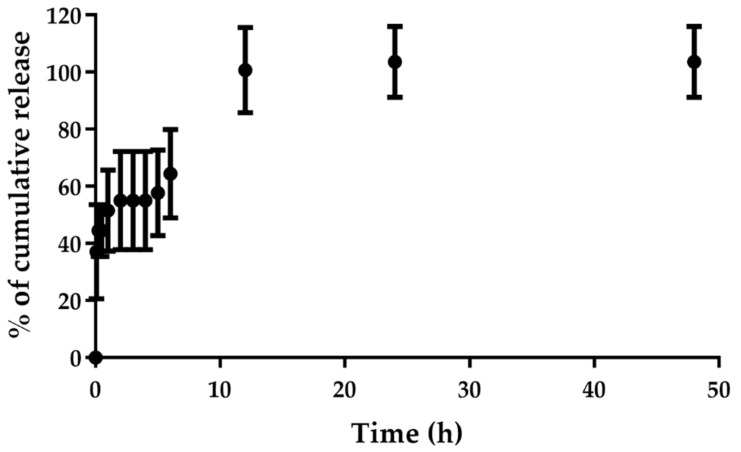
Cumulative release of C_8_ from AS1411-gold nanoparticles performed in 20 mM KPi and 100 mM KCl for 48 h (*n* = 3). Samples were collected at different time points (0 min, 5 min, 15 min, 30 min, 1 h, 2 h, 3 h, 4 h, 5 h, 6 h, 12 h, 24 h and 48 h).

We investigated the release profile of C_8_ from the AS1411-AuNPs in a buffer containing KPi (20 mM) and KCl (100 mM) over a period of 48 h. As seen in Figure 3, C_8_ was almost fully released from the nanoparticles during the first 12 h, suggesting that the drug can be efficiently released to the cancer cells. These results can be regarded as favorable, since the final goal is to produce a topical formulation to apply in the region of interest. Thus, the rapid release of the compound can be promoted in the region that we want to deliver it.

**Figure 4:**
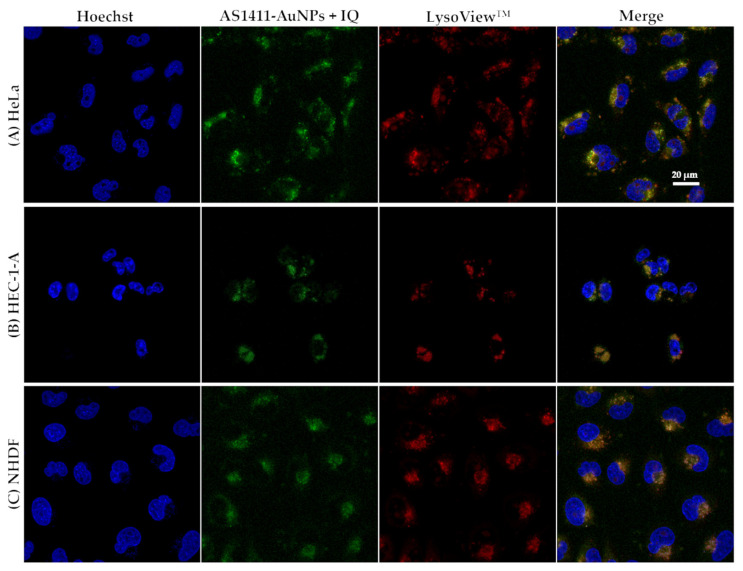
Confocal fluorescence images of (**A**) HeLa, (**B**) HEC-1-A, and (**C**) NHDF cell lines incubated with AS1411-gold nanoparticles with IQ (AS1411-AuNPs + IQ; green) for 2 h at 37 °C. Lysosomes are stained with LysoViewTM shown in red. Cell nuclei are stained with Hoechst 33342 (blue). Scale bar: 20 µm.

Then, we performed cellular studies for free compounds and the AS1411-AuNPs, carrying or not the anticancer drugs. Initially, the cellular uptake of free IQ was studied in HeLa, HEC-1-A and NHDF cells by confocal microscopy. As observed in Figure S5, HeLa and HEC-1-A cells were able to better internalize free IQ, which is consistent with the fact that IQ already has some selectivity for cancer cells, which could be related to its modulatory effect in the NF-κB signaling pathway, which plays a fundamental role in tumorigenesis [35,36]. The same effect was not seen with C_8_, which was internalized by all the cell lines (Figure S6). However, when IQ was added to aptamer-conjugated nanoparticles, there was a higher uptake of the nanoparticles by the cancer cell lines relative to the free IQ and some greater internalization by NHDF cells (compare Figure S5C and Figure 4C). This is probably due to the fact that normal cells also internalize AS1411, as previously described [12].

**Figure 5:**
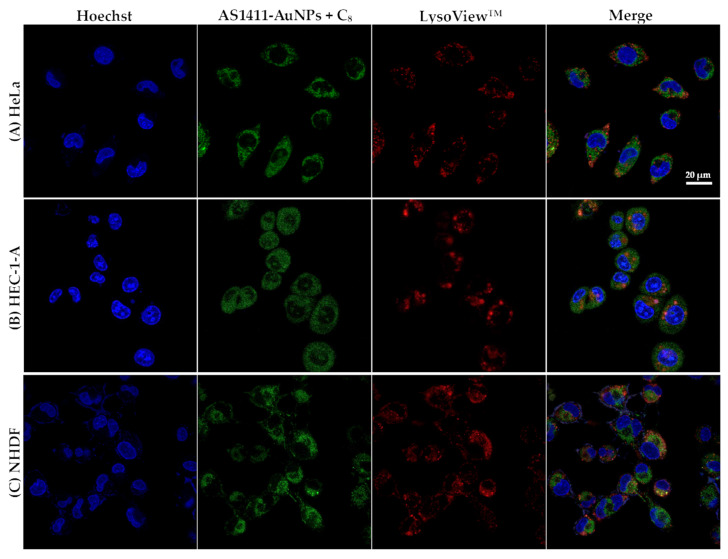
Confocal fluorescence images of (**A**) HeLa, (**B**) HEC-1-A, and (**C**) NHDF cell lines incubated with AS1411-gold nanoparticles with C_8_ (AS1411-AuNPs + C_8_; green) for 2 h at 37 °C. Lysosomes are stained with LysoViewTM shown in red. Cell nuclei are stained with Hoechst 33342 (blue). Scale bar: 20 µm.

To determine whether the improved drug delivery was due to lysosomal entrapment, we used LysoViewTM 540 to observe lysosome localization. As observed in Figure 4B,C, the AS1411-AuNPs colocalized with the lysosomotropic dye in HEC-1-A and NHDF cells, respectively (orange staining in the merge image). In HeLa cells, this colocalization was less pronounced, and a green coloration was observed in the merged image (Figure 4A). Similar distribution of AS1411-AuNPs loaded with C_8_ was observed in all cell lines (Figure 5), with a green coloration, suggesting that the nanoparticles were outside the lysosomal compartment. Nevertheless, the conjugation with AS1411-AuNPs conferred a higher selectivity to C_8_.

Previously, it has been reported that after lysosomal compartmentalization, the AS1411 aptamer can be eliminated from cells [12]. The involvement of this mechanism would not have been observed in the previous images, as cells were constantly cultured in the same medium containing the AS1411-AuNPs. Taking this into account, a new experiment involving fresh medium replacement (without any addition of AS1411-AuNPs with IQ or C_8_) was performed. The NHDF cells showed a residual internal fluorescence signal with the AS1411-AuNPs loaded with IQ (Figure S7C) or C_8_ (Figure S8C). However, in HeLa cells (Figures S7A and S8A) and HEC-1-A cells (Figures S7B and S8B) the fluorescence from the nanoparticles remains inside the cells. Thus, it seems as though the efflux mechanism could remove nanoparticles from normal cells while the cancer cells retained the nanoparticles. These results are in accordance with previous ones obtained by Malik et al., where AS1411-AuNPs accumulated at higher levels in cancer cells than in normal cells [21].

**Figure 6:**
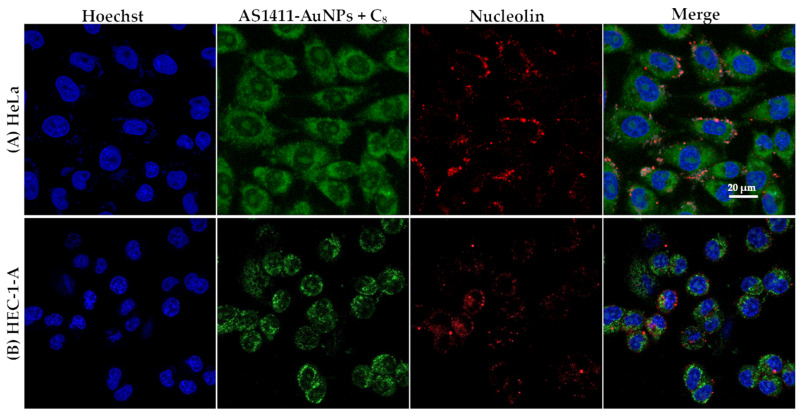
Confocal fluorescence images of (**A**) HeLa and (**B**) HEC-1-A cells incubated with AS1411-gold nanoparticles with C_8_ for 15 min at 37 °C. Nucleolin is stained with AlexaFluor 647^®^ (red), C_8_ emits green fluorescence. Cell nuclei are stained with Hoechst 33342 (blue). Scale bar: 20 µm.

**Figure 7:**
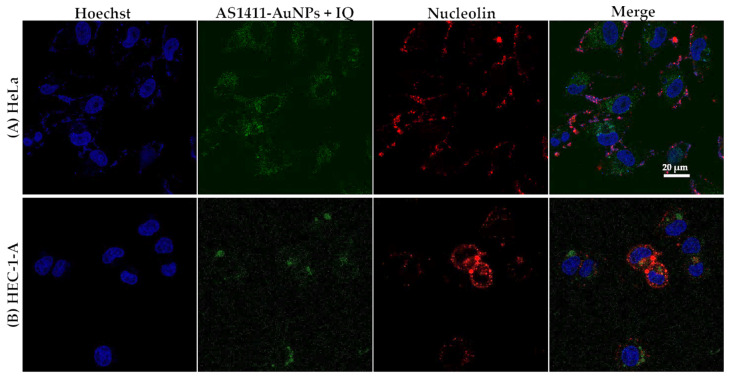
Confocal fluorescence images of (**A**) HeLa and (**B**) HEC-1-A cells incubated with AS1411-gold nanoparticles with IQ for 15 min at 37 °C. Nucleolin is stained with AlexaFluor 647^®^ (red), IQ emits green fluorescence. Cell nuclei are stained with Hoechst 33342 (blue). Scale bar: 20 µm.

The internalization mechanism of the aptamer AS1411 is likely to be different in cancer cells from in normal cells, because HeLa and HEC-1-A cells present nucleolin on their surfaces [29]. As was the case with free aptamer [23], AS1411-AuNPs loaded with C_8_ were internalized by nucleolin-positive cells (Figure 6). After 15 min of incubation, almost all nanoparticles were internalized by the cells and localized in the cytoplasm compartment. Similar results were obtained with IQ, as shown in Figure 7.

In normal cells (Figure S9), nucleolin is not overexpressed and the cells did not internalize the compound as quickly as the cancer cells, showing a lower green fluorescence than the malignant cell lines.

Although AS1411 has been reported to bind to nucleolin, there have been additional reports that the initial uptake of AS1411 is nucleolin-independent [12]. This mechanism could be associated with the absence of colocalization of AS1411-AuNPs with this protein in the confocal studies, since the nanoparticles were only in contact with the HeLa and HEC-1-A cells for 15 min. Other endocytic mechanisms were also proposed by Reyes-Reyes et al. for the internalization of AS1411, such as micropinocytosis [12]. Contrarily, normal cells internalize AS1411 through other mechanisms, which lead to endosomal entrapment or lysosomal degradation, and consequently, cell death is not induced [12]. Similar mechanisms may be happening in our experiments with AS1411-AuNPs carrying potential anticancer drugs, which can benefit from these cancer selective mechanisms. Overall, it is possible that nucleolin recognition may localize aptamers on the cells surface, followed by generic macropinocytosis, which would avoid endosomal capture and overall make this targeting mechanism more efficacious for functional drug delivery.

**Figure 8:**
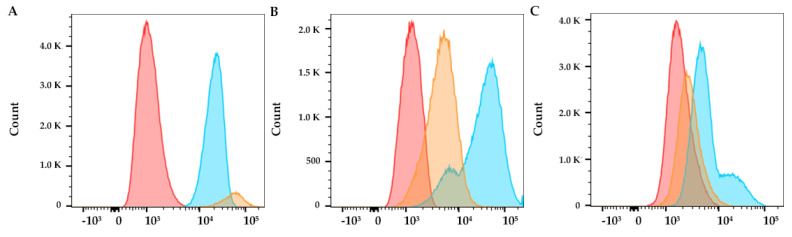
Flow cytometry analysis after free C_8_ (blue) or AS1411-AuNPs + C_8_ (15:1, yellow) incubation for 24 h in (**A**) HeLa cancer cells, (**B**) HEC-1A cancer cells, and (**C**) NHDF non-malignant cells. Control condition (only cells) are indicated by red coloration.

Flow cytometry studies were also performed to evaluate cellular uptake in normal and cancer cells, by following the native fluorescence of the compound. C_8_ assembled with AS1411-AuNPs show higher internalization in HeLa cancer cells relative to normal cells, as shown in Figure 8. Therefore, the AS1411-AuNPs were able to provide C_8_ with selectivity towards the cervical cancer cell line and should improve the overall therapeutic index. In HEC-1-A cells, the internalization of C_8_ is higher, which could suggest that AS1411-AuNPs are more selective for HeLa cervical cancer cells. The differences in fluorescence levels, when compared with the confocal microscopy experiment (Figure 5), could be related to the different incubation times (2 h vs. 24 h).

**Figure 9:**
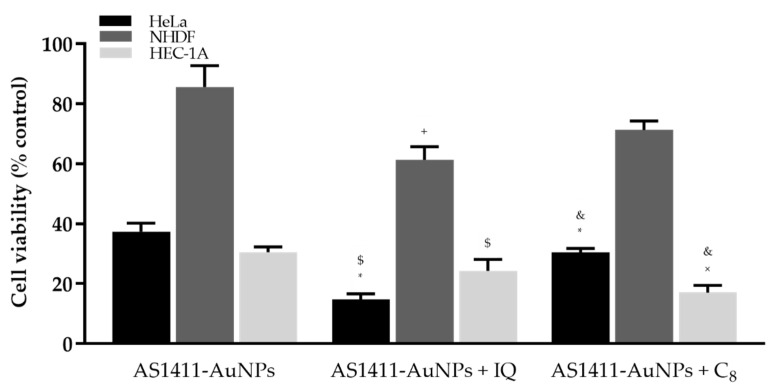
Percentage of viable HeLa, HEC-1-A and NHDF cells after incubation with AS1411-gold nanoparticles (AS1411-AuNPs) (15 µM), AS1411-AuNPs with IQ (15:5) or AS1411-AuNPs with C_8_ (15:1) for 3 days. * *p* < 0.05 relative to AS1411-AuNPs in HeLa cells; $ *p* < 0.05 relative to AS1411-AuNPs with IQ in NHDF cells; + *p* < 0.05 relative to AS1411-AuNPs in NHDF cells; & *p* < 0.05 relative to AS1411-AuNPs with C_8_ in NHDF cells; × *p* < 0.05 relative to AS1411-AuNPs in HEC-1-A cells.

The AS1411 aptamer exhibits antiproliferative activity against various cancer cell lines but requires several days for anticancer action to be observed. Previous results showed that the use of AS1411-nanoparticles can improve AS1411 uptake and decrease the time required for its action [21]. We also find that nanoparticles at a final concentration of 15 µM, as seen in Figure 9, are highly selective. In normal cells, they are not toxic, presenting a cellular viability of 85.00 ± 7.01%. In contrast, their toxicity is much higher in cancer cells, with a cellular viability of 37.34 ± 2.83% in HeLa cells and 30.50 ± 1.76% in HEC-1-A cells. Previously, it was found that at least 5 days of treatment was necessary to observe the antiproliferative effect of AS1411 [14]. The present results were obtained after 3 days of treatment, demonstrating that the covalent bonding of AS1411 to AuNPs was able to decrease the time required for aptamer function.

IQ is usually used in lesions caused by low-risk HPC, but it has been suggested that it may also have anticancer effects in malignant cells resulting from high-grade HPV infections [27]. Therefore, we evaluated the effect of IQ on the cellular viability of normal and cancer cells, in order to understand what nanoparticle concentrations might be used to treat various HPV lesions. As observed in Figure S2A,C,E, cervical cancer cells have a higher sensitivity to IQ (with an IC_50_ value of 4.22 µM) compared to normal cells (IC_50_ of 7.41 µM) and to endometrial cancer cells (IC_50_ of 25.77 µM). IQ at a concentration of 5 µM gave cell viabilities in HeLa, HEC-1-A and NHDF cells of 31.09 ± 3.10%, 36.33 ± 3.31% and 49.22 ± 3.24%, respectively. The higher toxicity in cancer cells is presumably due to their higher cellular uptake of IQ, as observed by confocal microscopy (Figure S5).

The in vitro cytotoxic effects of the complex AS1411-AuNPs with IQ were finally tested on HeLa and HEC-1-A vs. NHDF cells, using an MTT assay. For the same 5 µM concentration of the drug, the AS1411-AuNPs with IQ (61.37 ± 4.33%, Figure 9) showed lower cellular toxicity in non-malignant cells than the free IQ (49.22 ± 3.24%, Figure S2E) (*p*-value = 0.0506). Additionally, the nanoparticles with IQ were also able to increase the cytotoxicity linked to IQ (14.70 ± 1.91%, Figure 9) in the cervical cancer line, relative to free IQ (31.09 ± 3.10%, Figure S2A) (*p*-value = 0.0046). Relative to the endometrial carcinoma cell line, AS1411-AuNPs with IQ (24.25 ± 3.90%, Figure 9) showed a tendency to improve the cytotoxicity of IQ alone (36.33 ± 3.31%, Figure S2C) (*p*-value = 0.0730). Briefly, the complex was able to improve selectivity for cancer cells by decreasing the toxic effect in the non-malignant cell line.

Similar experiments were performed with C_8_, which has previously been demonstrated to be highly toxic in both normal and cancer cell lines [23,29,30]. In accordance with these observations, the IC_50_ values of free C_8_ incubated with HeLa, HEC-1-A and NHDF cells for three days were 0.26 µM (Figure S2B), 0.10 µM (Figure S2D) and 0.27 µM (Figure S2F), respectively. In previous studies we used C_8_ at 1 µM [23,29,30], so the same concentration was considered in this work to perform the association of C_8_ to AS1411-AuNPs (15 µM) based on the supramolecular strategy. This concentration is high enough to elicit significant cell toxicity while still being suitable for observing any changes induced by the ligand complexation to the aptamer. Then, the resulting complex was incubated with HeLa, HEC-1-A and NHDF cells and their viability was evaluated to verify whether the AS1411-AuNPs could also improve the cancer selectivity of this drug. As observed in Figure 9 and Figure S2, the AS1411-AuNPs significantly improved the cancer selectivity of C_8_. In fact, 1 µM of free C_8_ strongly comprised the viability (<10%) of all cell lines to a similar extent; when carried by nanoparticles, C_8_ is considerably more cytotoxic against tumoral cells than normal ones, as also observed for those carrying AS1411-AuNPs.

In brief, IQ addition to the AS1411-AuNPs seems to affect NHDF cells (85.66 ± 7.01% vs. 61.37 ± 4.33%; *p* = 0.0146), while on HEC-1A cells it has a minor impact (30.50 ± 1.76% vs. 24.25 ± 3.90%; *p* = 0.1944); however, on HeLa cells it appears to have a larger effect (37.34 ± 2.83% vs. 14.70 ± 1.91%; *p* < 0.0001). In the case of C_8_ addition to AS1411-AuNPs, it seems to be safer for NHDF cells (85.66 ± 7.01% vs. 71.31 ± 2.98%; *p* = 0.0604), exerting a minor impact on HeLa (37.34 ± 2.83% vs. 30.45 ± 1.31%; *p* = 0.0331), and was more effective on HEC-1A cells (30.50 ± 1.76% vs. 17.00 ± 2.48%; *p* = 0.0044). These results suggest that the conjugation of AS1411-AuNPs with C_8_ is more appropriate for use in the endometrial carcinoma cell line, while conjugation with IQ is more appropriate for application in cervical cancer cells.

Overall, MTT assay demonstrated that besides acting as a drug carrier, the AS141-AuNPs also presents an intrinsic therapeutic effect and can potentiate the drug anticancer effects.

The promising nanoparticles were formulated for topical application and their potential to permeate vaginal tissue was evaluated using Franz cells. Porcine vaginal tissue was used in Franz cells, and AS1411-AuNPs with C_8_ were incorporated in topical formulations (hydroxypropyl methylcellulose gels) containing either 8% or 10% propylene glycol. As observed in Table 2, both formulations containing the AS1411-AuNPs loaded with C_8_ were retained in the vaginal tissue after 24 h but were not detected in the receptor chamber, as determined by HPLC-FLD analysis at different time points. However, these studies were performed only for 24 h, so the formulation may be able to permeate the tissue and reach the receptor chamber after longer exposure times; the formulation cannot permeate the tissue and reach the receptor chamber. Moreover, there is still a large amount of active compound in the donor chamber after 24 h (Table 2). Nevertheless, the nanoparticle formulation containing 10% of propylene glycol seems to be better retained in the tissue compared to the 8% formulation. (Table 2). These results suggest that the viscosity of the formulation could influence the retention of the nanoparticles in the vaginal tissue.

## 4. Conclusions

In this study, drug-loaded AS1411-AuNPs were successfully produced and characterized in terms of particle size, zeta potential and in vitro drug release. The drug-loaded AS1411-AuNPs presented small sizes (about 15–20 nm), negative surface charge, high stability, and an adequate in vitro drug release profile. The antiproliferative effects against HeLa cervical cancer and HEC-1-A endometrial carcinoma cells were also determined, and the particles were able to improve the cancer selectivity of drugs such as IQ and C_8_, as demonstrated by confocal microscopy, flow cytometry and MTT assays. IQ-AS1411-AuNPs toxicity in HeLa and HEC-1-A cells and therapeutic index relative to normal cells were both improved, as demonstrated by a MTT assay. C_8_-AS1411-AuNPs also showed cancer accumulation of C_8_ and a decrease in cellular viability (30.5% in HeLa and 17.0% in HEC-1-A cancer cells and 71.3% in normal cells). These results are consistent with the fact that in normal cells, aptamer-conjugated nanoparticles are constantly removed by efflux or exocytosis mechanisms, in accordance with confocal and cytometry results. This effect is further enhanced by the fact that normal cells present low levels of nucleolin, and internalized complexes in cancer cells can be entrapped in endosomes or suffer from lysosomal degradation, as suggested by staining with LysoView^TM^.

The aptamer-conjugated nanoparticle is an improved strategy for IQ, especially in cervical cancer cells, since it enhances the anticancer potential and selectivity of IQ itself, by decreasing the viability of cancer cells (HeLa with ~15% and HEC-1A with ~24%) compared to normal NHDF cells (~86%). However, with C_8_ the effect was different from that obtained with AS1411-AuNPs+IQ. In a manner comparable to the results obtained with the AS1411-AuNPs per se, C_8_-loaded nanoparticles had a similar effect on HeLa cells (only less 7% than the cell viability obtained with AS1411-AuNPs alone; ~37% to ~30%), they had a lower effect in the viability of NHDF cells (about 15% less than the result obtained with AS141-AuNPs alone; ~86% to ~71%), and they had a promising effect on HEC-1A cells, improving the toxic effect of the nanoparticles in this cancer cell line (reducing the result obtained with the AS1411-AuNPs by almost 50%; ~30% to ~17%). These results suggest that the different drug-loaded AS1411-AuNPs are more effective in determined cell lines. For instance, AS1411-AuNPs+IQ are more promising for application in cervical cancer, while C_8_ show greater promise in the endometrial carcinoma cell line. The aptamer-conjugated nanoparticles were also formulated for application in vaginal lesions. Permeation studies using porcine vaginal tissue demonstrated the efficacy of the gel formulation in retaining and accumulating drugs in the vaginal epithelia.

Overall, the results achieved in this study provide evidence that aptamer-conjugated nanoparticles may prove to be suitable for cancer-specific drug delivery and as promising therapeutic agents for gynecologic cancers.

## Figures and Tables

**Table 1:** Hydrodynamic diameter and Zeta potential of the nanoparticles.

	Hydrodynamic Diameter (nm)	Zeta Potential (mV)
Gold Nanoparticles	12.00 ± 1.20	−24.00 ± 1.01
AS1411-Gold Nanoparticles	15.76 ± 1.64	−25.84 ± 4.06
AS1411-Gold Nanoparticles with IQ	21.16 ± 2.68	−40.43 ± 3.12
AS1411-Gold Nanoparticles with C_8_	18.30 ± 2.53	−41.30 ± 3.21

**Table 2:** C_8_ concentrations (ng/mL) present in the different compartments after the permeation study on porcine vaginal tissue (24 h in Franz cells) after treatment with formulation containing AS1411-AuNPs with C_8_. The results represent the mean and standard deviation of 3 tissue Franz cell experiments. n.d.—the compound was not observed in the proper retention time in the HPLC-FLD analysis.

	8% Formulation	10% Formulation
Donor Chamber	3.97 ± 2.81	1.12 ± 0.80
Vaginal Tissue	0.625 ± 0.08	0.78 ± 0.51
Receptor Chamber	n.d.	n.d.

## Data Availability

The data presented in this study are available on request from the corresponding author.

## Supplementary Materials

The following are available online at https://www.mdpi.com/article/10.3390/cancers13164038/s1, Figure S1: Chromatogram of C_8_ at 100 ng/mL; Figure S2: Dose-response data of cell viability measured by MTT assay after incubation of Imiquimod or C_8_; Figure S3: TEM image of citrate gold nanoparticles; Figure S4: Histogram of TEM results; Figure S5: Confocal fluorescence images after IQ incubation; Figure S6: Confocal fluorescence images after C_8_ incubation; Figure S7: Confocal fluorescence images after AS1411-AuNPs with IQ incubation; Figure S8: Confocal fluorescence images after AS1411-AuNPs with C_8_ incubation; Figure S9: Confocal fluorescence images of NHDF treated with AS1411-AuNPs and stained with anti-nucleolin antibody.
