# Supplementary material for: Aptamer-Functionalized Gold Nanoparticles for Drug Delivery to Gynecological Carcinoma Cells
Source: Cancers (Basel). 2021 Aug 11;13(16):4038. doi: 10.3390/cancers13164038 (PMC8391588; doi:10.3390/cancers13164038)
Supplement: Supplementary file 1 [file cancers-13-04038-s001.zip › cancers-1295676-supplementary.pdf]

cancers

MDPI

Supplementary Materials

Aptamer-Functionalized Gold Nanoparticles for Drug Delivery to Gynecological Carcinoma Cells

Jessica Lopes-Nunes [1], Ana S. Agonia [2], Tiago Rosado [1,3], Eugénia Gallardo [1], Rita Palmeira-de-Oliveira [1,2,4], Ana Palmeira-de-Oliveira [1,2], José Martinez-de-Oliveira [1], José Fonseca-Moutinho [1,5], Maria Paula Cabral Campello [6,7], Artur Paiva [8,9,10], António Paulo [6,7], Alexa Vulgamott [11], Andrew D. Ellignton [11], Paula A. Oliveira [12], Carla Cruz [1,*]

[1] CICS-UBI Centro de Investigação em Ciências da Saúde, Universidade da Beira Interior, Av. Infante D. Henrique, 6200-506 Covilhã, Portugal
[2] Labfit - HPRD Health Products Research and Development, Lda, Edifício UBIMEDICAL Estrada Municipal 506, 6200-284 Covilhã, Portugal
[3] C4 - Cloud Computing Competence Centre, UBIMedical, Universidade da Beira Interior, EM506, 6200-284, Covilhã, Portugal
[4] Center for Neuroscience and Cell Biology, University of Coimbra, Universidade de Coimbra, Rua Larga, 3004-504 Coimbra, Portugal
[5] Centro Hospitalar Universitário Cova da Beira, Covilhã, Portugal
[6] Centro de Ciências e Tecnologias Nucleares, Instituto Superior Técnico, Universidade de Lisboa, Estrada Nacional 10 (km 139,7), 2695-066 Bobadela LRS, Portugal
[7] DECN -Departamento de Engenharia e Ciências Nucleares, Instituto Superior Técnico, Universidade de Lisboa, Portugal
[8] Unidade de Gestão Operacional em Citometria, Centro Hospitalar e Universitário de Coimbra (CHUC), Coimbra, Portugal
[9] CIMAGO/iCBR/CIBB, Faculdade de Medicina da Universidade de Coimbra, Coimbra, Portugal
[10] Instituto Politécnico de Coimbra, ESTESC-Coimbra Health School, Ciências Biomédicas Laboratoriais, Coimbra, Portugal
[11] Department of Molecular Biosciences, The University of Texas at Austin, Austin, TX 78712, USA
[12] Centre for Research and Technology of Agro Environmental and Biological Sciences (CITAB), Inov4Agro, University of Trás os Montes and Alto Douro (UTAD), Quinta de Prados, 5000 801 Vila Real, Portugal
* Correspondence: Carla Cruz (carlacruz@fcsaude.ubi.pt). Tel: +351275329097; Fax: +351275329099.

Index

Figure S1	3
Figure S2	4
Figure S3	5
Figure S4	5
Figure S5	6
Figure S6	7
Figure S7	8
Figure S8	9
Figure S9	10

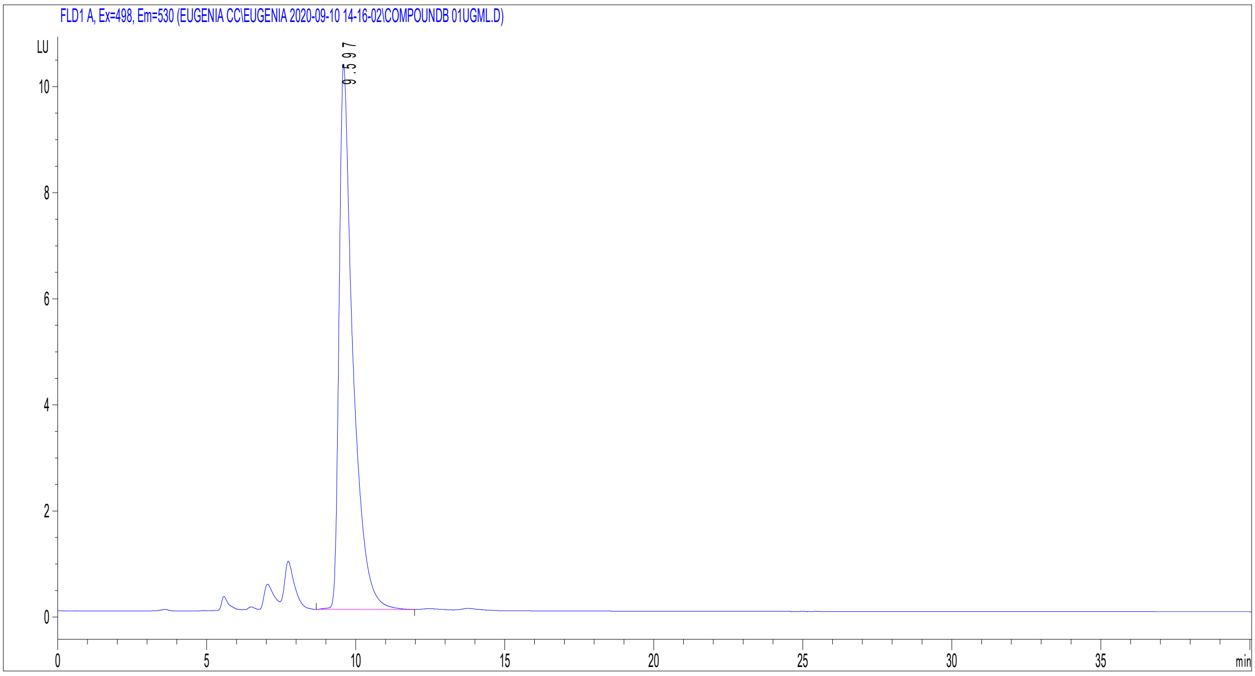

Figure S1. Chromatogram of C_8 at 100 ng/mL.

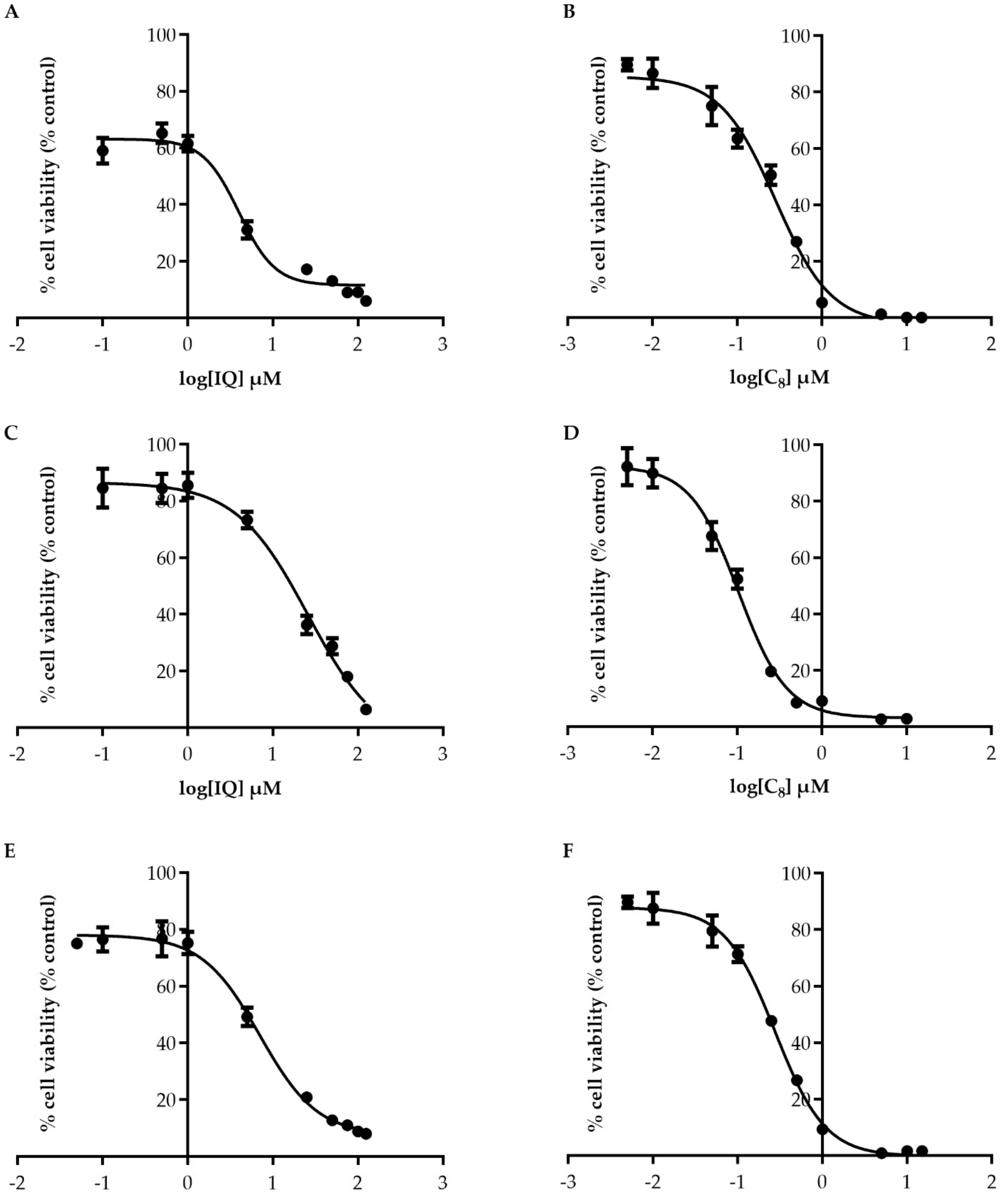

Figure S2. Dose-response data of cell viability measured by MTT assay after incubation of Imiquimod or C_8;(A) HeLa, (C) HEC-1-A and (E) NHDF cells treated with Imiquimod (B) HeLa, (D) HEC-1-A and (F) NHDF cells treated with C_8 for 3 days.

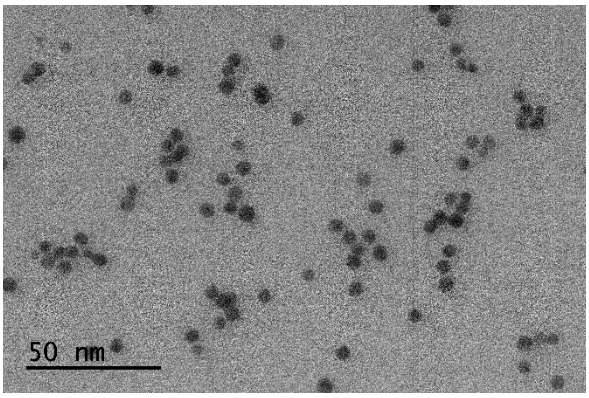

Figure S3. TEM image of citrate gold nanoparticles.

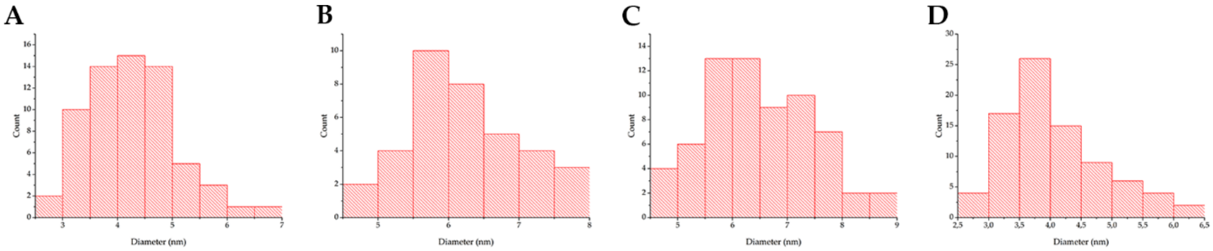

Figure S4. Histograms of the nanoparticles size determined in TEM images of (A) citrate gold nanoparticles, (B) AS141-AuNPs, (C) AS1411-AuNPs with IQ and (D) AS1411-AuNPs with C_8.

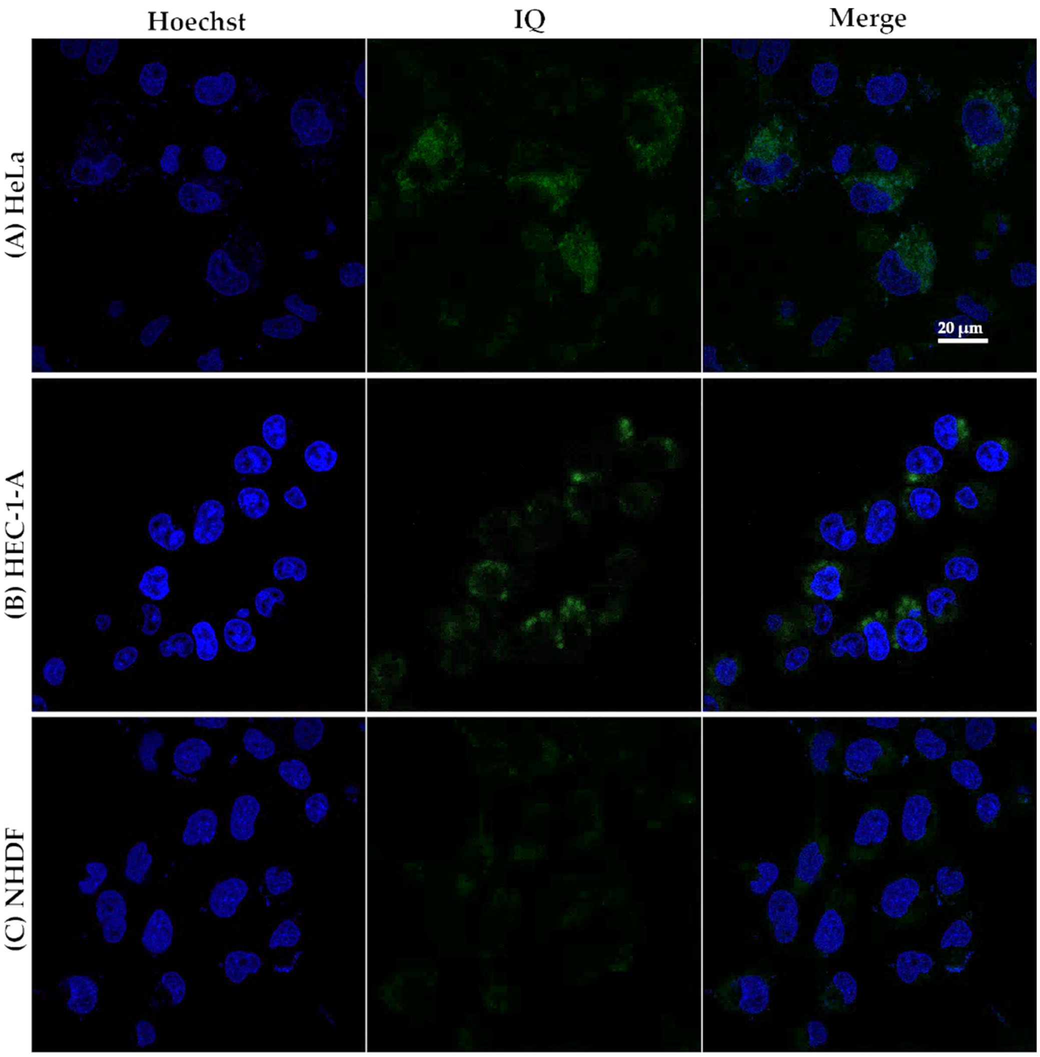

Figure S5. Confocal fluorescence images after IQ incubation; (A) HeLa, (B) HEC-1-A and (C) NHDF cell lines incubated with IQ (green) for 24 h at 37 °C. Cell nuclei are stained with Hoechst 33342 (blue). Scale bar: 20 μm.

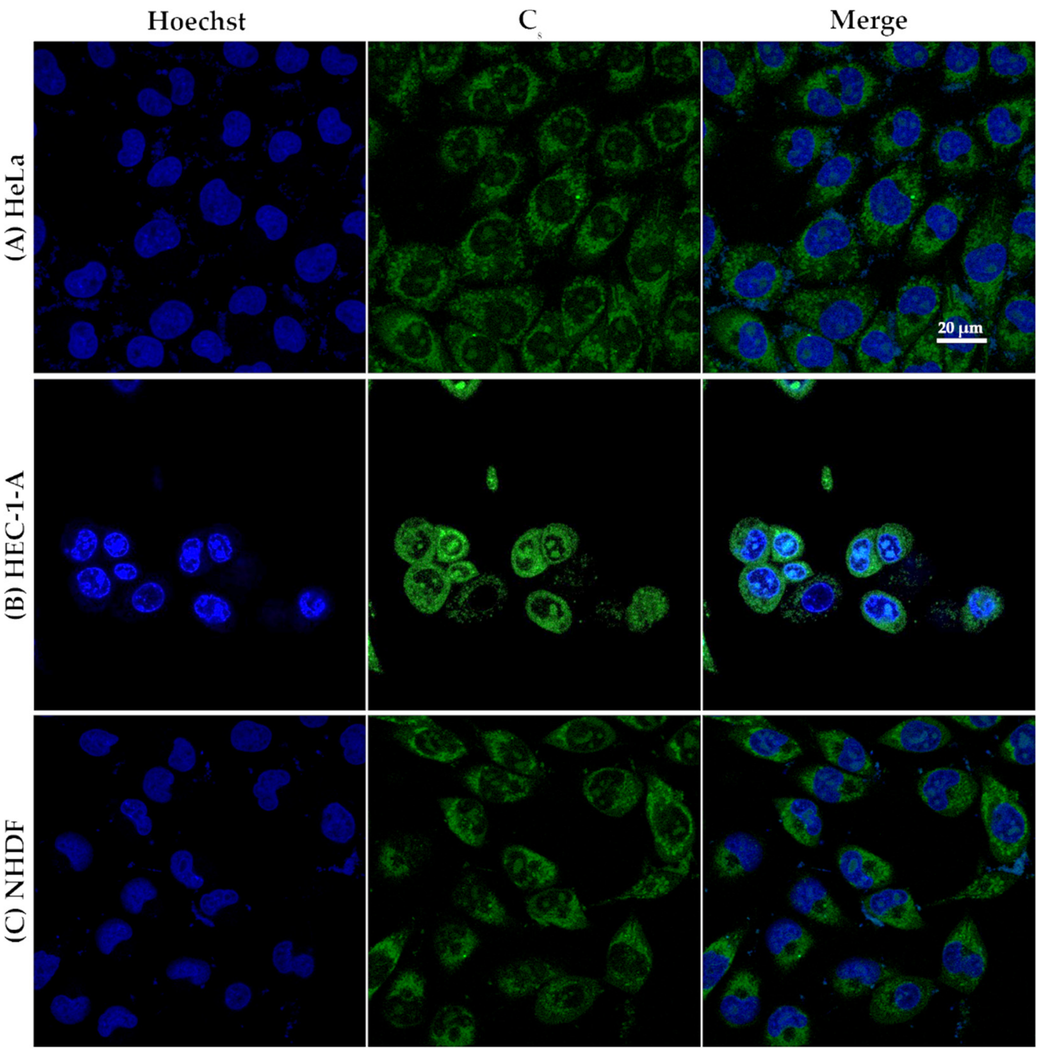

Figure S6. Confocal fluorescence images after C_8 incubation; (A) HeLa, (B) HEC-1-A and (C) NHDF cell lines incubated with C_8 (green) for 24 h at 37 °C. Cell nuclei are stained with Hoechst 33342 (blue). Scale bar: 20 μm.

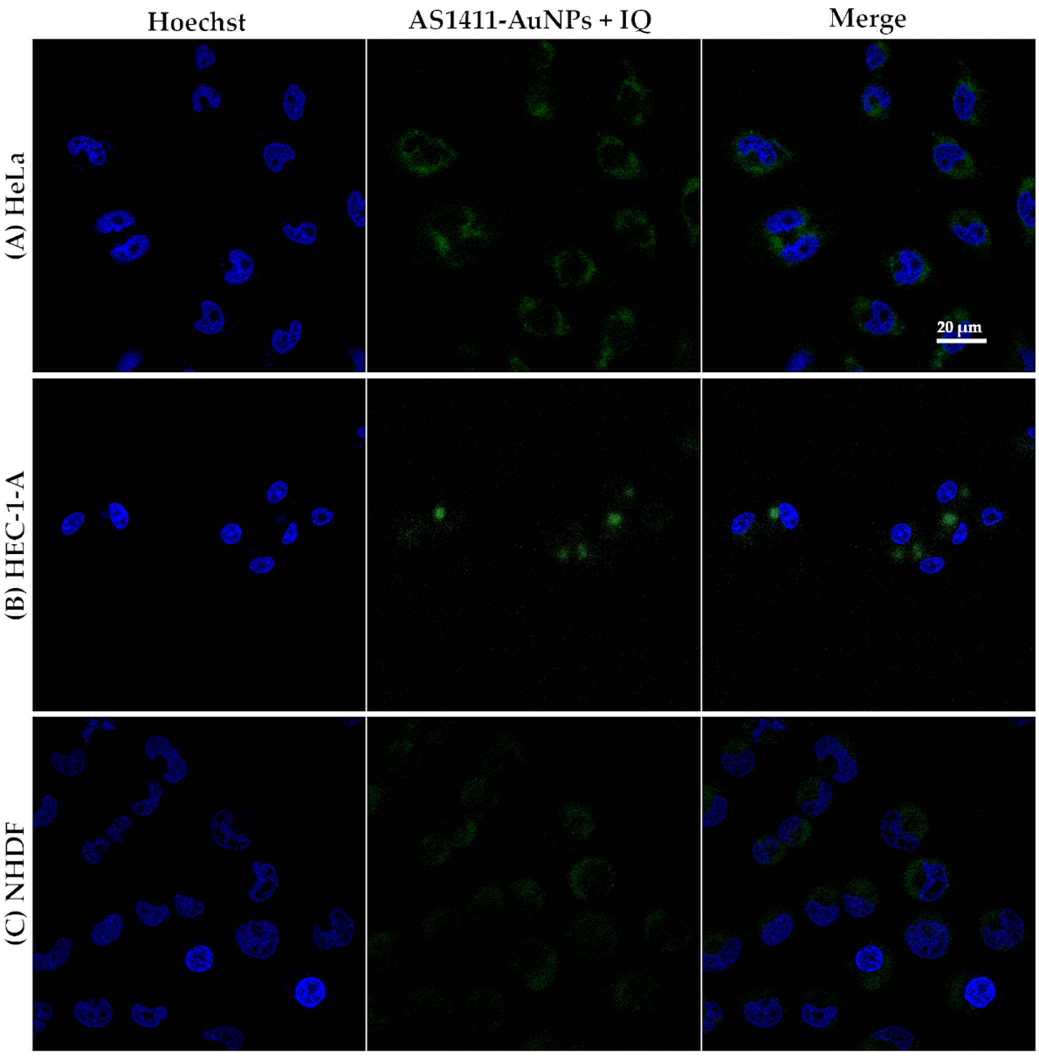

Figure S7. Confocal fluorescence images after AS1411-AuNPs with IQ incubation; (A) HeLa, (B) HEC-1-A and (C) NHDF cell lines incubated with AS1411-AuNPs with IQ (green) for 24 h at 37 °C and, then, medium was replaced by new one without treatment. Cell nuclei are stained with Hoechst 33342 (blue). Scale bar: 20 μm.

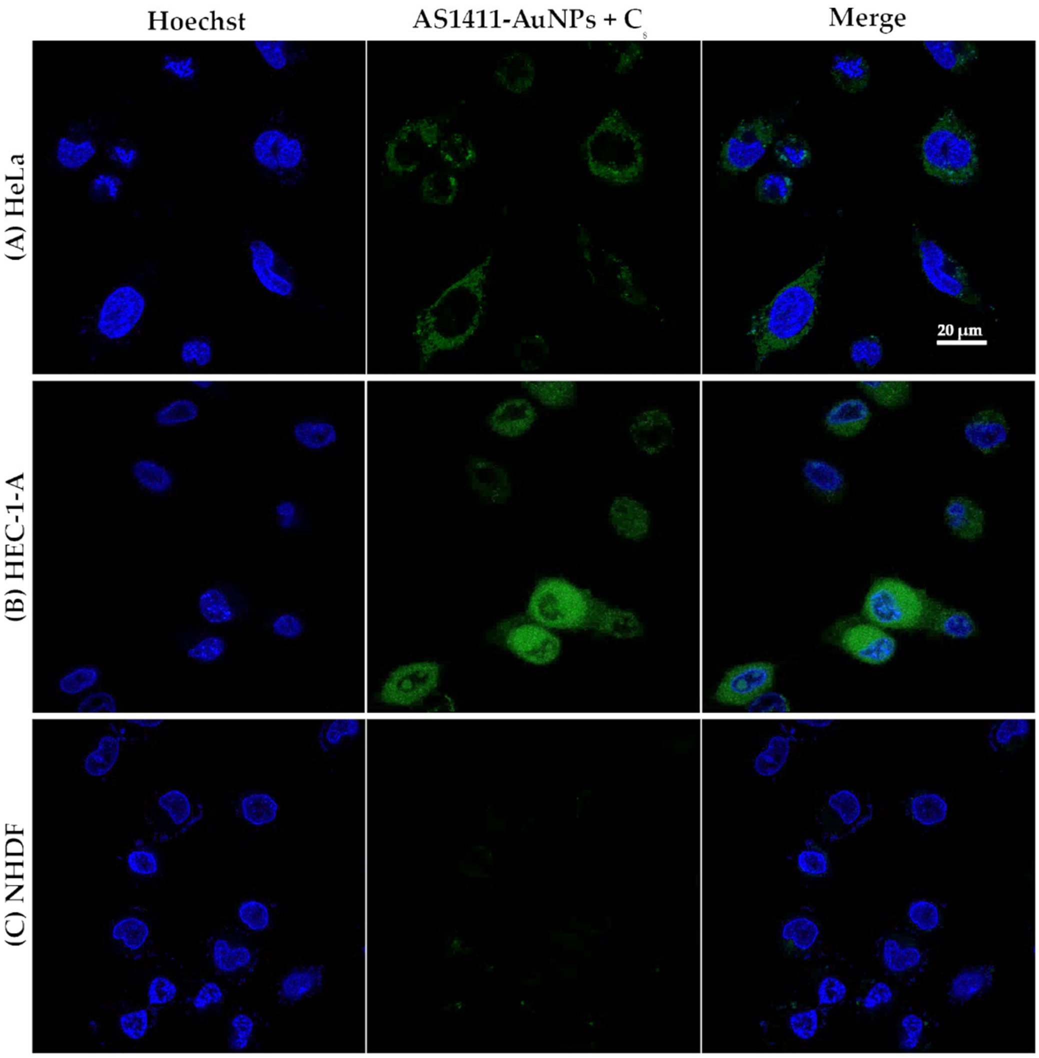

Figure S8. Confocal fluorescence images after AS1411-AuNPs with C_8 incubation; (A) HeLa, (B) HEC-1-A and (C) NHDF cell lines incubated with AS1411-AuNPs with C_8 (green) for 24 h at 37 °C and, then, medium was replaced by new one without treatment. Cell nuclei are stained with Hoechst 33342 (blue). Scale bar: 20 μm.

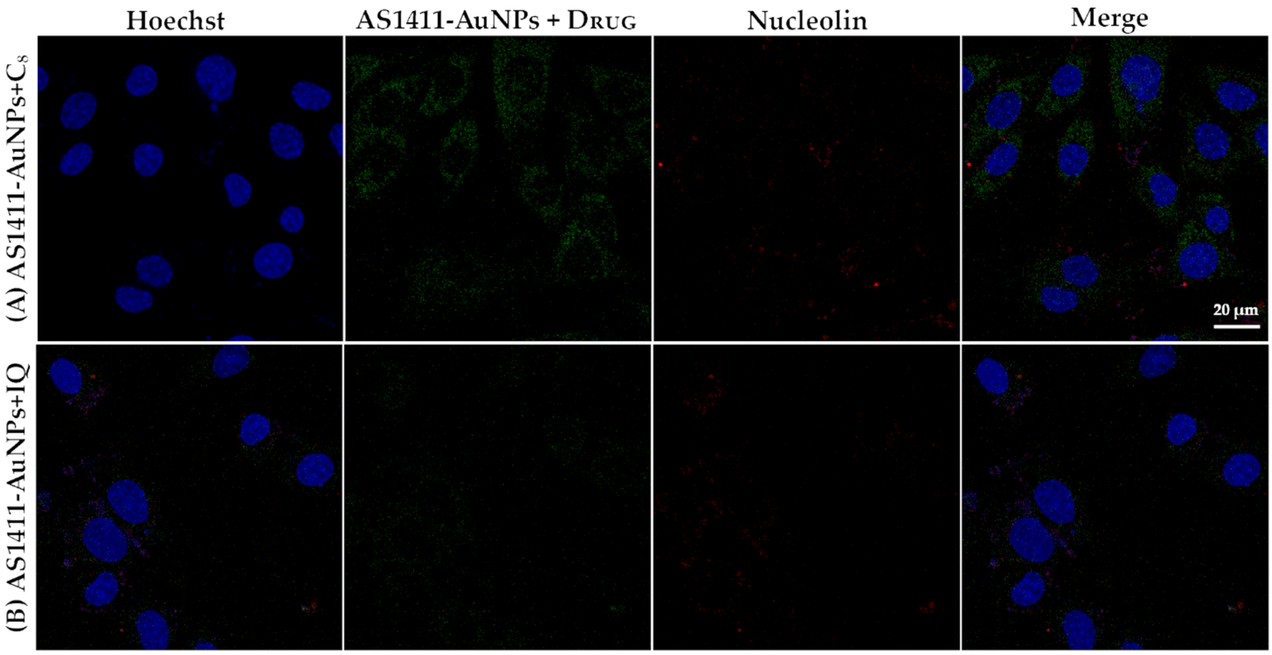

Figure S9. Confocal fluorescence images of NHDF cells incubated with (A) AS1411-gold nanoparticles with C_8 or (B) AS1411-gold nanoparticles with IQ for 15 min at 37 °C. Nucleolin is stained with AlexaFluor 647® (red), C_8 and IQ emits green fluorescence. Cell nuclei are stained with Hoechst 33342 (blue). Scale bar: 20 μm.
